# Supplementary material for: Innovative Galenic Formulation of Prussian Blue Tablets: Advancing Pharmaceutical Applications
Source: Pharmaceuticals (Basel). 2025 Oct 17;18(10):1568. doi: 10.3390/ph18101568 (PMC12566741; doi:10.3390/ph18101568)
Supplement: Supplementary file 1 [file pharmaceuticals-18-01568-s001.zip › pharmaceuticals-3895106-supplementary.pdf]

Innovative Galenic Formulation of Prussian Blue Tablets: Advancing Pharmaceutical Applications Supplementary Materials

Borja Martínez-Alonso [1], Guillermo Torrado Durán [1] , Norma S. Torres Pabón [1] and M. Ángeles Peña Fernández [1,*]

[1] Department of Biomedical Sciences, Faculty of Pharmacy, University of Alcalá (UAH), Campus Universitario, Crta. Madrid – Barcelona km. 33.600, 28771 Alcalá de Henares (Madrid), Spain; borja.matineza@uah.es

* Correspondence: angeles.pena@uah.es; University of Alcalá (UAH) Campus Universitario, Crta. Madrid – Barcelona km. 33.600, 28771 Alcalá de Henares (Madrid), Spain; Tel.: +34 918854725; Fax: +34 918854658

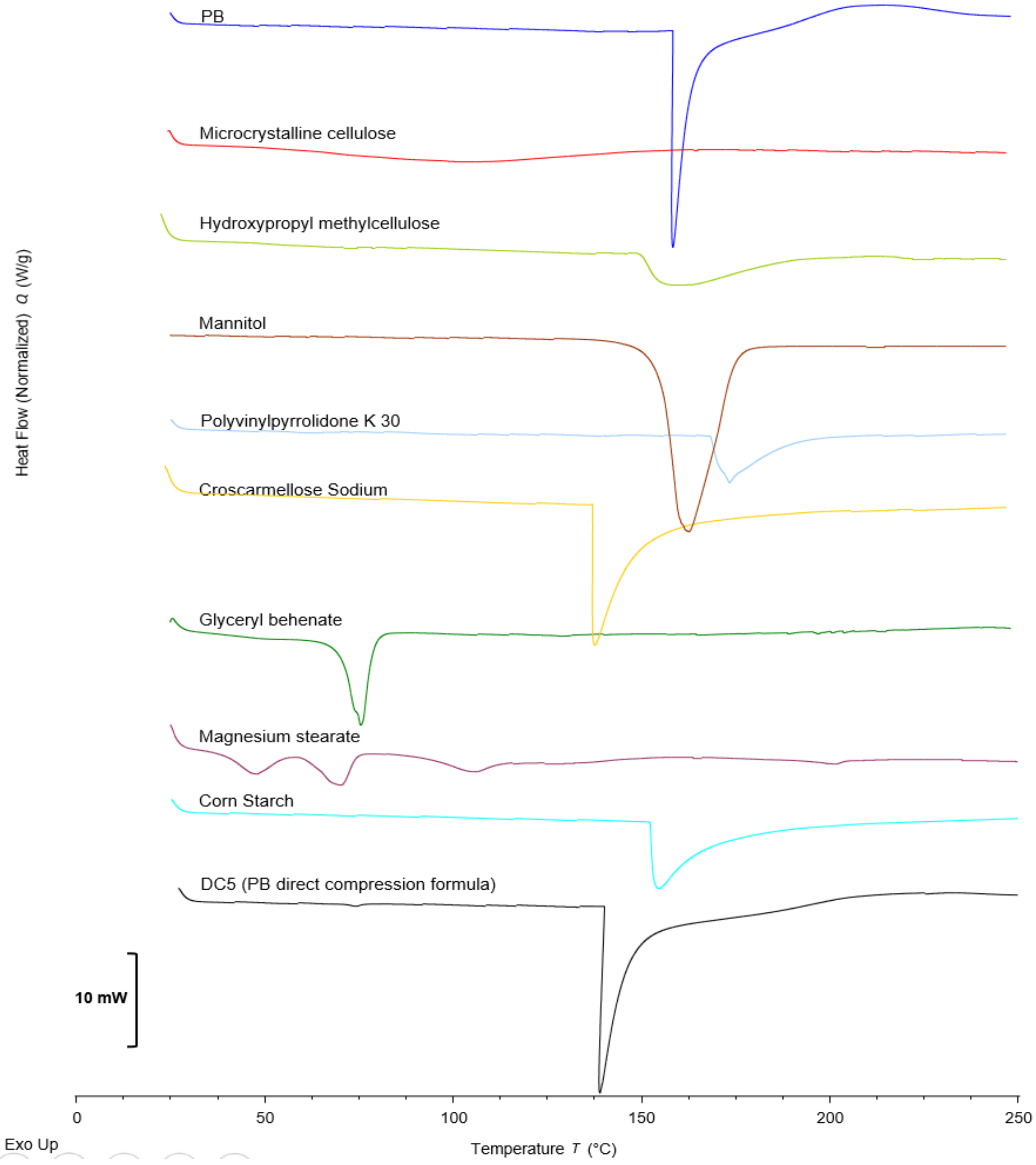

Figure S1. DSC-thermograms of the PB raw material, all single excipient samples and the direct compression blending DC5.

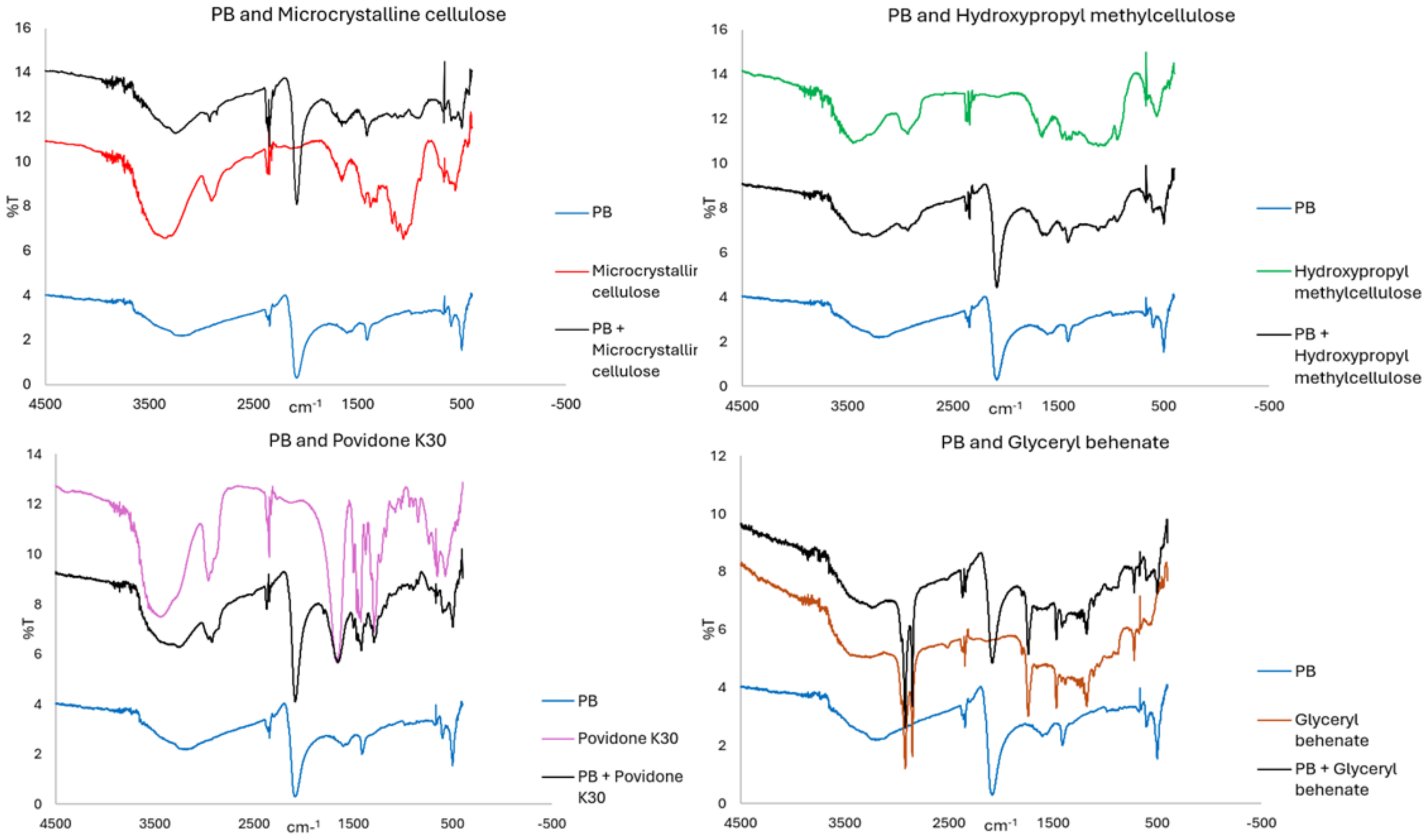

Figure S2. FTIR spectra of Prussian blue (PB), each excipient of the DC5 formulation, and their respective binary mixtures with PB.

Table S1. Step and overall yields by manufacturing route (means of available batches).

Route / Batch	Blend handling / Mixing (%)	Granulation (%)	Drying (%)	Compaction–milling (%)	Screening target-fraction (%)	Compression (%)	Overall tablet yield (%)
DC5 (development)	99	-	-	-	-	95–96	>95
DC5 (pilot; stability lots)	≈99	-	-	-	-	≈99	≈98
WG (galenic/legacy equipment)	≈99	97	99	-	97	97	≈90
DG (index batch)	99	-	-	≈89	≈88	≈90	≈70

1. DC1–DC4: exploratory screening formulations; yields not recorded.
2. WG screening mass balance:): >1.00 mm 1%; 0.60–1.00 mm 90%; 0.30–0.60 mm 7%; <0.30 mm 2%. Target-fraction yield = 90% + 7% (all the 0.6-0.3 fraction was added as it was inferior to the 20% of 1.0-0.6 fraction) = 97%.
3. DG screening mass balance (index batch): >1.00 mm 4%; 0.60–1.00 mm 73%; 0.30–0.60 mm 18%; <0.30 mm 5%. Target-fraction yield = 73% + (0.20 × 18%) = 87.6%, (≈88).
4. DG overall (≈70%) estimated as: 0.99 × 0.89 × 0.876 × 0.90 ≈ 0.70.
5. DC5 pilot overall (≈98%) reflects reduced compression losses in scaled pilot runs.

Table S2. PB tablets (500 mg) - Finished product specifications (applied at release and on stability unless stated)

Test / Attribute	Method & Reference	Stage	Acceptance Criteria
Description	Visual (RFE. general)	Release & Stability	Blue tablets, round, biconvex; without breaks or visible defects.
Assay (content of PB, as Fe)	Validated ICP method (see Section 3.8.2); acceptance aligned with ICH Q6A practice	Release & Stability	95.0–105.0 % of label claim (data-driven per development/stability).
Uniformity of dosage units	Ph. Eur. 2.9.40 (Content Uniformity)	Release	AV ≤ 15.0 (L1 = 15.0). If AV > 15.0 (n=10), test n=30; final AV ≤ 15.0 and no individual < (1–0.25)·M nor > (1+0.25)·M.
Mean mass	RFE 2.9.5 (uncoated; mean ≥ 250 mg)	Release & Stability	665.0–735.0 mg (±5 % of 700 mg). Max: ≤ 2/20 outside ±5 % and none outside ±10 %.
Hardness (resistance to crushing)	Tablet hardness tester; n = 10 (Section 3.8.2)	Release & Stability	NLT 39.2 N (informative robustness criterion; used for trending alongside friability).
Friability	RFE 2.9.7 (n = 10; 100 revs, 25 rpm)	Release & Stability	NMT 1.0 % loss of mass.
Disintegration	RFE 2.9.1 (water, 37 ± 2 °C; n = 6)	Release & Stability	NMT 15 min.
% Weight loss (TGA)	TGA (GA55; 25→360°C, 20°C·min^{-1}; N_2 40 mL·min^{-1})	Release & Stability	NLT 25.0 %.
Cs-binding capacity	In-vitro method (Section 3.8.2: efficacy subsection)	Release & Stability	NLT 89 %; on stability: NLT (initial – 10 %).

1. Assay (PB equivalent via ICP-Fe with TGA-based hydration correction): 95.0–105.0 % of label claim [24].
2. Uniformity of dosage units: Ph. Eur. 2.9.40 equations and staging (AV, k, M) as stated in the manuscript Methods [50].
3. Mean mass: RFE 2.9.5, uncoated tablets, mean ≥ 250 mg (criteria inserted in Methods and applied here) [44].
4. Hardness: not a pharmacopoeia limit test; used as informative mechanical attribute. Criterion NLT 39.2 N corresponds to the development target ensuring robustness: trending alongside friability [46,47].
5. Friability: Ph. Eur./RFE 2.9.7; method described in Section 3.8.2 (Pharmatest PTF-E) [48].
6. Disintegration: Ph. Eur./RFE 2.9.1 (water, 37 ± 2 °C); method described in Section 3.8.2 (Pharmatest PTZ-E) [45].
7. % Weight loss (TGA) & Cs-binding: methods as per Sections 3.8.2 / Efficacy; same acceptance used during stability (see Tables 8–10).

References

24. International Conference on Harmonisation of Technical Requirements for Registration of Pharmaceuticals for Human *Use. Stability Testing of New Drug Substances and Products Q1A(R2);* ICH Harmonised Tripartite Guideline: Geneva, Switzerland, 2003.
50. 2.9.40 Uniformity of dosage units, In: *European Pharmacopoeia*, 11th ed., Council of Europe, Strasbourg, France, 2023.
44. 2.9.5 Uniformidad de Masa de las Preparaciones Presentadas en Dosis Únicas, In: *Real Farmacopea Española*, 6ª ed., Ministerio de Sanidad y Consumo, Madrid, Spain, 2023.
46. 2.9.8 Resistencia de los comprimidos a la rotura, In: *Real Farmacopea Española*, 6ª ed., Ministerio de Sanidad y Consumo, Madrid, Spain, 2023.
47. Eraga, S.; Baridakara, Z. J.; Iwuagwu, M. A comparative investigation of the disintegrant efficiency of *Musa paradisiaca*. *J. Pharm. Bioresour.* **2016**, 13, 114. https://doi.org/10.4314/jpb.v13i2.7
48. 2.9.7 Friabilidad de los comprimidos no recubiertos, In: Real Farmacopea Española, 6ª ed., Ministerio de Sanidad y Consumo, Madrid, Spain, 2023.
45. 2.9.1 Disgregación de comprimidos y cápsulas, In: *Real Farmacopea Española*, 6th ed.; Ministerio de Sanidad y Consumo: Madrid, Spain, 2023.
